# Synergistic Anti-Inflammatory Effects of Pomegranate Peel–Hawthorn Combinations in Ulcerative Colitis: Network Pharmacology Prediction and Experimental Validation

**DOI:** 10.3390/cimb47040243

**Published:** 2025-04-01

**Authors:** Shouqing Zhang, Quanyuan Qiu, Mengzhen Yuan, Jiajia Yu, Weiwei Gao, Xi Wang, Zhen Liu, Peng Yu, Cen Xiang, Yuou Teng

**Affiliations:** China International Science and Technology Cooperation Base of Food Nutrition/Safety and Medicinal Chemistry, State Key Laboratory of Food Nutrition and Safety, Tianjin University of Science and Technology, Tianjin 300457, China

**Keywords:** ulcerative colitis, pomegranate peel, hawthorn, ellagic acid, maslinic acid

## Abstract

Ulcerative colitis (UC) is a chronic inflammatory bowel disease characterized by complex pathogenesis involving dysregulated immunity and gut microbiota imbalance, demanding innovative therapeutic strategies. This study investigates the synergistic therapeutic potential of pomegranate peel–hawthorn combinations and their active constituents (ellagic acid and maslinic acid) through an integrative approach combining network pharmacology, in vitro/in vivo experiments, and gut microbiota analysis. Network pharmacology identified 61 shared therapeutic targets (*p* < 0.05 for pathway enrichment) and revealed complementary mechanisms: pomegranate peel primarily modulated AGE-RAGE/PI3K-Akt pathways, while hawthorn targeted IL-17/NF-κB signaling. Experimental validation demonstrated potent synergistic anti-inflammatory effects (combination index < 1), with optimal combinations reducing nitric oxide production by 52.35% (herbal extracts, *p* < 0.05) and 74.4% (active monomers, *p* < 0.05). In DSS-induced UC mice, combinatorial therapies significantly suppressed pro-inflammatory cytokines (TNF-α: 204.78 vs. 446.52 pg/mL in UC group, *p* < 0.05; IL-6: 33.19 vs. 64.86 pg/mL, *p* < 0.05), restored colonic SOD activity (72.31 vs. 50.10 U/mg·prot in UC group, *p* < 0.01), and alleviated histopathological damage, outperforming monotherapeutics. Gut microbiota analysis revealed the recovery of α-diversity indices and normalized Bacteroidota/Bacillota ratios. Mechanistically, the combinations suppressed MAPK/NF-κB signaling cascades, reducing p-p38/p38 (*p* < 0.01 vs. UC group) and p-ERK1/2/ERK1/2 (*p* < 0.01 vs. UC group) phosphorylation. These findings establish that pomegranate peel–hawthorn formulations exert multi-modal therapeutic effects through the synergistic inhibition of pathways, mitigation of oxidative stress, and restoration of the microbiota, offering a scientifically validated approach for UC management rooted in traditional medicine principles.

## 1. Introduction

Ulcerative colitis (UC), a chronic inflammatory bowel disease, is characterized by progressive colorectal mucosal injury manifesting as bloody diarrhea, abdominal pain, and increased bowel frequency [1,2,3,4]. Its pathogenesis involves genetic susceptibility, immune dysregulation, microbiota alterations, and epithelial barrier dysfunction [5], with chronic inflammation elevating colorectal cancer risk [6]. Epidemiologically, UC has shown a rising global incidence, particularly in Asian populations approaching Western rates (12–26/100,000) in recent decades [7,8,9].

Molecular mechanisms center on the interplay between inflammation and oxidative stress, featuring the dominance of pro-inflammatory cytokines (TNF-α, IL-1β, and IL-6) over anti-inflammatory IL-10 [10,11,12,13,14]. The NF-κB pathway orchestrates inflammatory responses and apoptosis, while MAPK/ERK/JNK signaling modulates these processes through cytokine interactions [15,16,17,18,19,20,21]. Concurrent gut microbiota alterations show reduced diversity with Firmicutes/Bacteroidetes depletion and Proteobacteria expansion [22,23,24], disrupting immune homeostasis [25]. Existing therapies (aminosalicylates, corticosteroids, and biologics) face limitations due to adverse effects [26,27,28,29], driving interest in herbal interventions, which are used by 40% of patients for their microbiota-modulating and barrier-protective potential.

Traditional Chinese medicine (TCM) classifies UC under “dysentery/diarrhea” disorders, demonstrating efficacy through anti-inflammatory, antioxidant, and immunomodulatory mechanisms [30,31]. Recently, attention has turned to pomegranate peel (SLP) and hawthorn (SZ) combinations. SLP and SZ are selected based on their unique pharmacological profiles and documented efficacy in inflammatory bowel disease (IBD). SLP, which is rich in polyphenols such as ellagitannins and ellagic acid, has been shown to attenuate dextran sulfate sodium-induced colitis in mice by modulating the gut microbiota and enhancing mucosal barrier function [32]. Additionally, SLP extracts have demonstrated the ability to reduce oxidative stress and inflammation in intestinal epithelial cells, highlighting their potential in UC therapy [33]. SZ, on the other hand, contains flavonoids and phenolic acids that not only inhibit pro-inflammatory cytokines but also promote the growth of beneficial gut bacteria such as Lactobacillus and Bifidobacterium, which are often depleted in UC patients [34]. These preclinical findings suggest that combining SLP and SZ could synergistically address multiple pathogenic pathways in UC.

SLP’s polyphenols (ellagitannins and ellagic acid) exhibit properties including NF-κB/MAPK pathway modulation, cytokine reduction, and mucosal healing through tight junction enhancement [35,36,37]. SZ’s flavonoids and phenolic acids similarly inhibit inflammatory signaling while promoting beneficial microbiota [38,39]. Their synergistic potential combines pathway regulation, antioxidant boosting, and microbiota modulation with improved safety profiles [40].

This study investigates the therapeutic mechanisms of pomegranate peel–hawthorn combinations and their active compounds (ellagic acid–hawthorn acid) using multimodal analyses of inflammatory pathways, oxidative responses, and microbiota dynamics.

## 2. Materials and Methods

### 2.1. Target Prediction and Protein–Protein Interaction (PPI) Network Construction

The pomegranate peel and hawthorn targets were predicted using the TCM Systematic Pharmacology Database (TCMSP; https://tcmsp-e.com/tcmsp.php (accessed on 21 March 2020)) and the Swiss Target Prediction Database (http://www.swisstargetprediction.ch/ (accessed on 25 March 2020)). All gene targets related to UC were obtained from the GeneCards database (https://www.genecards.org/ (accessed on 25 March 2020)) and the DisGeNET database (https://www.disgenet.org/ (accessed on 25 March 2020)). Standardized targets were obtained from the UniProt database (https://tcmsp-e.com/index.php (accessed on 27 March 2020)). The overlapping genes and a Venn diagram of pomegranate peel, hawthorn, and UC targets were obtained from the online Venn map platform (https://bioinfogp.cnb.csic.es/tools/venny/ (accessed on 27 March 2020)). The gene targets were further imported into the STRING database (https://cn.string-db.org/(accessed on 27 March 2020)), in order to explore the interactions between the known and predicted proteins. The topological parameters in the PPI network were analyzed using Cytoscape 3.9.0.

### 2.2. Enrichment of Gene Ontology (GO) Terms and Kyoto Encyclopedia of Genes and Genomes (KEGG) Pathways

We imported the obtained potential targets of pomegranate peel and hawthorn for UC treatment into the target gene name list through the DAVID database (https://david.ncifcrf.gov/home.jsp (accessed on 31 March 2020)), restricting the species to humans. GO enrichment analysis and KEGG pathway annotation analysis were performed on the potential targets, and the results were processed using the Microbiotics online platform (https://www.bioinformatics.com.cn/ (accessed on 31 March 2020)) to screen the important signaling pathways of pomegranate peel and hawthorn in UC.

### 2.3. High-Performance Liquid Chromatography (HPLC) Assay

The hawthorn extract, pomegranate peel extract, hawthorn acid standard (>98%), and ellagic acid standard (>98%) were accurately weighed and separately dissolved in acetonitrile to prepare 1 mg/mL test solutions. After filtration with a 0.22 μm membrane, the samples were stored in HPLC vials. An analysis was performed on a C18 column at 25 °C with an injection volume of 10 μL, a flow rate of 0.8 mL/min, and a detection wavelength of 254 nm. For the hawthorn extract and hawthorn acid standard, we used mobile phases A (aqueous phase) and B (acetonitrile), with gradient elution as follows: 15%–10% A over 0–12 min; 10%–15% A over 12–20 min. For pomegranate peel extract and ellagic acid standard, we used mobile phases A (aqueous phase) and B (acetonitrile), with gradient elution as follows: 95%–80% A over 0–5 min, 80%–70% A over 5–15 min, and 70%–60% A over 15–25 min. A blank run confirmed that the retention times of the ellagic acid and ursolic acid were free from interference.

### 2.4. Cell Lines and Culture Conditions

RAW264.7 cell lines were purchased from the Peking Union Medical College Cell Resource Center (Beijing, China). RAW264.7 cells were grown at 37 °C and a 5% CO_2_ atmosphere in growth medium (Dulbecco’s modified Eagle medium (DMEM, Gibco; Beijing; China)) supplemented with 10% fetal bovine serum (FBS, Gibco; Beijing; China) and 1% penicillin–streptomycin. The cells were passaged every 2 d. The cell culture medium supernatant was used to produce a RAW264.7 cell suspension from the fusion culture, and a hemocytometer was used to determine the cell concentration.

### 2.5. Cytotoxic Activity Assay

Compound toxicity to cells was detected using an MTT assay. In brief, RAW264.7 cells were plated on 96-well plates at 1 × 10^5^ cells per well in a 100 μL medium. After 24 h, the incubation was continued after adding the tested compounds. After 24 h of incubation, 10 μL of MTT (5 mg/mL) was added to each well, and the plates were further incubated for 4 h. Then, DMSO was added to the plate to dissolve formazan. A microplate reader (Tecan, Grödig, Austria) was used to measure the absorbance at 492/630 nm.

### 2.6. Determination of Nitric Oxide in Cell Supernatant

To collect cell supernatant for the nitric oxide (NO) assay, RAW264.7 cells were plated in 24-well plates as 1 × 10^5^ cells per well in 0.5 mL of medium. After 24 h of incubation, the medium was discarded, and the cells were washed with phosphate-buffered saline (PBS) (1 mL); after which, 0.5 mL of serum-free medium was added to each well. Subsequently, 2.5 μL of lipopolysaccharide (LPS) was added to each well for 1 h. The blank group did not need to be added. After 1 h, 2.5 μL of the tested compounds was added to the dosing wells for 24 h of incubation. After 24 h of incubation, the cell supernatant was collected into a 1.5 mL EP tube and centrifuged at 4000 rpm for 10 min. Afterward, the centrifuged supernatant was tested using nitric oxide assay kits from the Nanjing Jiancheng Institute of Biotechnology (Nanjing, China).

### 2.7. Enzyme-Linked Immunosorbent Assay

The RAW264.7 cell treatment method was the same as described in the NO test method. TNF-α, IL-1β, IL-6, and IL-10 in the cell supernatants were analyzed with ELISA kits from the Nanjing Jiancheng Institute of Bioengineering (Nanjing, China) according to the manufacturer’s instructions.

### 2.8. Cell Intracellular Reactive Oxygen Species (ROS) Assay

The level of intracellular reactive oxygen species (ROS) was detected using the ROS Assay Kit (Solebo Technology Co., Ltd., Beijing, China). After drug treatment, RAW264.7 cells were collected and incubated in a serum-free medium containing DCFH-DA (10 μM) in the dark for 30 min. The cell fluorescence signal was assessed using flow cytometry (Accuri, Lexington, MA, USA).

### 2.9. Animal Models

Male C57BL/6 mice (6–8 weeks) were purchased from Changsheng Biotechnology Co., Ltd. (Liaoning, China; permit number SYXK (Tianjin), 2018-0001]). All animal procedures were performed according to the Guidelines for Care and Use of Laboratory Animals of Tianjin University of Science and Technology and approved by the Animal Ethics Committee of Tianjin University of Science and Technology (Reg. No. of Experimental Facilities Certification SYXK (津滨) 2023-0007; date, 20 November 2022). All mice were housed at about 25 °C under a 12 h light–12 h dark cycle with free access to food and water and were adaptively fed for 5 days. After acclimation for 5 days, the mice were randomly divided into 10 groups according to weight: blank control group (0.9% saline 200 μL * day), model group (4% *w*/*v* DSS by gavage, 9 d), curcumin group (50 mg/kg * day), sulfasalazine group (50 mg/kg * day), pomegranate peel group (1.5 kg/kg * day), hawthorn group (1.5 kg/kg * day), pomegranate peel with hawthorn group (1.5 kg/kg * day), ellagic acid group (50 mg/kg * day), hawthorn acid group (50 mg/kg * day), and ellagic acid combined with hawthorn acid group (50 mg/kg * day). The entire experimental cycle comprised 14 days. The blank control group was injected with 200 μL of 0.9% saline via gavage for 9 days, while the other 9 groups were injected with 200 μL of 4% DSS solution via gavage for 9 days. The blank control and DSS model groups were given 0.5% CMC-NA via gavage for 5 days starting on day 10, and the remaining 8 groups were given the drug via gavage for 5 days and then finished. After the last administration, all mice were starved and treated for 12 h. Blood was taken from the femoral artery, and the cervical vertebrae were dislocated and dissected. The blood was allowed to stand at room temperature for 2 h and then centrifuged at 4000 r/min for 10 min/2 times at 4 °C. The supernatant was the serum, and the serum was stored in 1 mL EP tubes at −80 °C. All organ tissues were stored at −80 °C. The colorectal length was weighed and then fixed or stored in 10% neutral formalin buffer or frozen for histopathological analysis and other biochemical parameters. The concentrations of aspartate aminotransferase (AST), alanine aminotransferase (ALT), and superoxide dismutase (SOD) were determined using commercial assay kits purchased from the Nanjing Jiancheng Institute of Biotechnology (Nanjing, China). The TNF-α, IL-6, and IL-1β levels were determined using an enzyme-linked immunoassay (ELISA) purchased from the Nanjing Jiancheng Institute of Biotechnology (Nanjing, China).

### 2.10. Western Blot

Cell or colon tissue samples were rapidly lysed with protein lyase. Protein samples were obtained after centrifugation and determined using the BSA method. Protein was separated using SDS-PAGE and transferred to a polyvinylidene fluoride (PVDF) membrane. After the membranes were blocked with 5% non-fat milk for 1 h, the PVDF membranes were incubated with the primary antibodies (1/1000) at 4 °C overnight. Subsequently, the membranes were incubated with the appropriate secondary antibodies for 1 h. α-Tubulin was used as an internal reference protein. A gray analysis of protein bands was quantified using ImageJ (V. 1.52a, Wayne Rasband National Institutes of Health, Kensington, MD, USA).

### 2.11. HE Staining

Colonic tissues were fixed with 10% neutral buffered formalin, embedded in paraffin, and sectioned into 4 μm thick sections. Hematoxylin and eosin (HE) staining was performed, and the results were observed via microscope.

### 2.12. S rRNA Sequencing of Gut Microbiota

Mouse feces were immediately loaded into 2.0 mL centrifuge tubes (3 feces per tube) and collected into liquid nitrogen for storage. Sample testing was entrusted to the Beijing Genomics Institution (BGI) to assess mouse gut microbiota using 16S ribosomal RNA amplification and V3–V4 region sequencing.

### 2.13. Statistical Analysis

All data were expressed as mean ± standard deviation. The results were analyzed using one-way analysis of variance (ANOVA), and significant differences were determined using Duncan’s test with GraphPad Prism 8.22 (San Diego, CA, USA). *p* < 0.05 was considered significant.

## 3. Results

### 3.1. Network Pharmacology Analysis and Experimental Validation of Active Components

**Figure 1:**
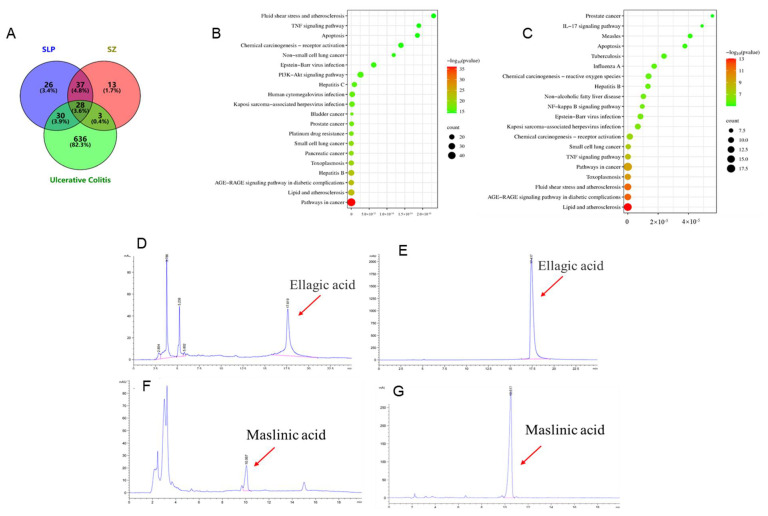
Network pharmacological and active ingredient analysis: VENNY analysis (**A**); Kyoto Encyclopedia of Genes and Genomes Analysis of pomegranate peel (**B**) and hawthorn (**C**); HPLC spectra of pomegranate peel extract (**D**); ellagic acid (**E**); hawthorn extract (**F**); and maslinic acid (**G**).

Through TCMSP database screening, we identified six bioactive components in pomegranate peel (Fritillaziebinol, ellagic acid, (+)-catechin, kaempferol, beta-sitosterol, and luteolin) and four in hawthorn (kaempferol, sitosterol, stigmasterol, and maslinic acid) with anti-inflammatory and antioxidant properties relevant to UC (Table S1). Kaempferol emerged as a shared component, suggesting synergistic potential. Bioinformatics analysis via Uniprot and disease databases (OMIM; TCGA) revealed 178 (pomegranate) and 96 (hawthorn) therapeutic targets, intersecting with 697 UC-related genes. Venn analysis identified 61 shared targets (Figure 1A), highlighting key mechanisms for UC intervention.

Cytoscape-based “herb component–target disease” networks (Figures S1 and S2) demonstrated robust connectivity between active components and UC targets. Pomegranate peel components interacted with apoptosis/inflammation regulators (TNF, AKT1, IL-6, and TP53), while hawthorn targeted inflammatory/apoptotic pathways (TNF, AKT1, PTGS2, and BCL2). PPI network analysis (Figures S3 and S4) confirmed TNF and AKT1 as common high-degree nodes, with pomegranate preferentially regulating ESR1/CASP3 and hawthorn modulating PPARG/BCL2.

GO enrichment (Figures S5 and S6) revealed the pomegranate peel’s involvement in apoptosis regulation and kinase signaling (nucleus/cytokine activity), whereas hawthorn influenced transcriptional regulation and redox processes (endoplasmic reticulum/oxidoreductase activity). KEGG pathway analysis (Figure 1B,C) showed that pomegranate peel predominantly modulated the AGE-RAGE, PI3K-Akt, and TNF pathways, while hawthorn targeted IL-17 and NF-κB signaling. Ellagic acid (pomegranate peel) and maslinic acid (hawthorn) emerged as unique components driving distinct pathway effects, suggesting complementary therapeutic mechanisms.

This network pharmacology approach systematically validated the multi-target mechanisms of pomegranate peel and hawthorn in UC treatment, identifying key components (ellagic acid and maslinic acid) and their pathway-specific actions (PI3K-Akt/NF-κB), supporting their synergistic potential for UC management.

The chemical composition of the pomegranate peel and hawthorn extracts was validated using high-performance liquid chromatography (HPLC). As shown in Figure 1D,E, ellagic acid in the standard solution exhibited a characteristic peak at approximately 17.5 min (Figure 1E). A corresponding peak was observed at the same retention time in the pomegranate peel extract (Figure 1D), confirming the presence of substantial ellagic acid in the pomegranate peels. Similar results were obtained for the hawthorn extract, validating the presence of its key active components, which were predicted using network pharmacology. These experimental findings corroborated the reliability of our earlier network pharmacology predictions regarding the active constituents of both herbs. Based on these results, we further investigated the therapeutic potential of combinatorial formulations, including pomegranate peel extract, hawthorn extract, combinations, and the paired active monomers (ellagic acid and maslinic acid) for UC treatment in subsequent experiments.

### 3.2. Anti-Inflammatory Efficacy and Synergistic Effects of Pomegranate Peel and Hawthorn Extracts with Active Constituents

**Figure 2:**
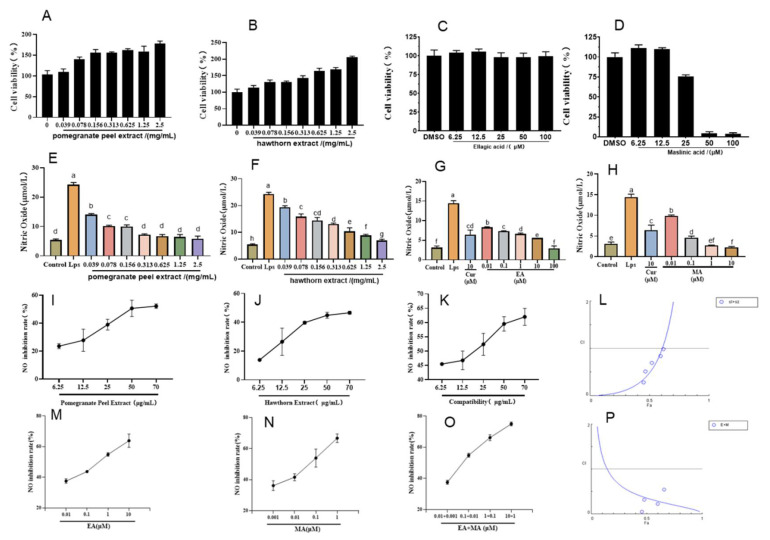
Activity of pomegranate peel extract and brake extract in vitro. Effects of pomegranate peel extract (**A**), hawthorn extract (**B**), ellagic acid (**C**), and maslinic acid (**D**) on the proliferative activity of RW264.7 cells; effects of pomegranate peel extract (**E**), hawthorn extract (**F**), ellagic acid (**G**), and maslinic acid (**H**) on NO content; NO inhibition rates of pomegranate peel extract (**I**), hawthorn extract (**J**), extract compatibility (**K**), ellagic acid (**M**), maslinic acid (**N**), and drug compatibility (**O**); CI value curves of extract compatibility (**L**) and drug compatibility (**P**). Different letters indicate *p* < 0.05.

The LPS-induced macrophage RAW264.7 was used to establish an in vitro cell inflammation model, and curcumin was selected as a positive drug to participate in the test. Before exploring the anti-inflammatory activity, we detected the effects of the pomegranate peel and hawthorn extracts, along with those of their active constituents (ellagic acid and maslinic acid), on RAW264.7 cytotoxicity using MTT assays. As shown in Figure 2A–D, cytotoxicity assessments revealed that both pomegranate peel and hawthorn extracts exhibited minimal toxicity toward RAW264.7 cells at concentrations of up to 2.5 mg/mL (Figure 2A,B). Ellagic acid demonstrated excellent safety profiles, maintaining high cell viability even at 100 μM (Figure 2C). In contrast, maslinic acid displayed concentration-dependent cytotoxicity, with cell viability dropping to 75.61%, 4.25%, and 3.73% at 25, 50, and 100 μM, respectively (Figure 2D). Based on these results, a non-toxic concentration range of 0–12.5 μM for maslinic acid was selected for subsequent experiments. These findings establish a safe dosage framework to further investigate the therapeutic potential of ellagic acid and maslinic acid in UC treatment.

Nitric oxide (NO) is a gaseous radical produced by inducible nitric oxide synthase (iNOS) in activated macrophages during LPS-induced inflammation, serving as a key inflammatory mediator. Our experiments evaluated the anti-inflammatory potential of crude extracts and active compounds by measuring NO production in LPS-stimulated RAW264.7 cells. As shown in Figure 2E,F, LPS stimulation significantly elevated NO levels to 24.25 μM. Pomegranate peel extract demonstrated potent NO inhibition, reducing levels to 5.81 μM at optimal concentrations, comparable to baseline (unstimulated cells). Similarly, hawthorn extract suppressed NO production to 6.91 μM, with both extracts exhibiting concentration-dependent anti-inflammatory effects. The calculated EC50 values were 54.6 μg/mL for pomegranate peel and 394.3 μg/mL for hawthorn, yielding a potency ratio of 1:8. Based on this ratio, combination therapies were tested at dose pairs of 70 + 560, 50 + 400, 25 + 200, 12.5 + 100, and 6.25 + 50 μg/mL (pomegranate peel–hawthorn). For active monomers, ellagic acid (EC_50_ = 0.32 μM) and maslinic acid (EC50 = 0.035 μM) displayed dose-dependent NO inhibition (Figure 2G,H), with a potency ratio of 10:1 (ellagic acid–maslinic acid). Combination treatments were subsequently administered at paired concentrations of 10 + 1, 1 + 0.1, 0.1 + 0.01, and 0.01 + 0.001 μM. These systematic dose–response analyses establish a quantitative framework for evaluating synergistic anti-inflammatory effects in subsequent UC therapeutic investigations.

The anti-inflammatory effects of pomegranate peel extract, hawthorn extract, and their combination were quantified by measuring nitric oxide (NO) inhibition rates at predetermined concentrations (Figure 2I–K). CompuSyn software analysis revealed combination index (CI) values < 1 across all tested dose pairs, confirming consistent synergistic interactions between the two herbal extracts. Notably, the combination of pomegranate peel crude extract (25 μg/mL) and hawthorn crude extract (200 μg/mL) demonstrated robust synergistic anti-inflammatory activity, achieving a NO inhibition rate of 52.35% with a CI value of 0.69. This ratio was selected for subsequent experiments due to its superior therapeutic potential. Similarly, dose-dependent NO inhibition was observed for ellagic acid (Figure 2L), maslinic acid (Figure 2M), and their combination (Figure 2N). Synergy analysis (Figure 2O) revealed that the combination of ellagic acid (10 μM) and maslinic acid (1 μM) exhibited the strongest synergistic effect (CI = 0.245), achieving the highest NO inhibition rate at 74.4% among all tested groups. This combination ratio was consequently chosen for further investigation. The consistent synergy observed in both crude extract and active monomer combinations underscores the therapeutic advantage of combinatorial formulations in mitigating LPS-induced inflammatory responses, validating the multi-target mechanisms predicted using network pharmacology.

### 3.3. Effects of Pomegranate Peel and Hawthorn Extracts, Along with Those of Their Active Constituents (Ellagic Acid and Maslinic Acid), on Inflammatory Factors in LPS-Induced RAW264.7

**Figure 3:**
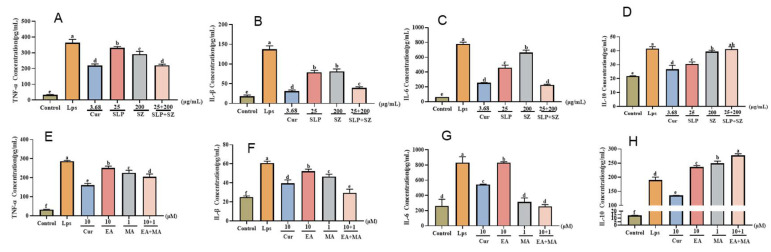
Effects of pomegranate peel and hawthorn extracts and co-administration on inflammatory factors in LPS-induced RAW264.7 cells. TNF-α, IL-1β, IL-6, and IL-10 concentrations of herb extracts (**A**–**D**) and active constituents (**E**–**H**). Cur, curcumin; SLP, pomegranate peel extract; SZ, hawthorn extract; EA, ellagic acid; MA, maslinic acid. Different letters indicate *p* < 0.05.

To comprehensively evaluate the anti-inflammatory effects beyond NO inhibition, we analyzed the modulation of key cytokines mediated using NF-κB signaling on LPS-stimulated RAW264.7 cells. As shown in Figure 3A–D, the combination of pomegranate peel and hawthorn extracts significantly suppressed pro-inflammatory cytokine secretion compared with monotherapy groups, reducing TNF-α, IL-1β, and IL-6 levels by 142.21 pg/mL, 98.33 pg/mL, and 554.66 pg/mL, respectively. Concurrently, the combination therapy enhanced anti-inflammatory cytokine IL-10 production, demonstrating dual regulatory effects on inflammatory homeostasis. These results indicate that the herbal combination exerts anti-inflammatory effects by simultaneously inhibiting pro-inflammatory mediators and promoting anti-inflammatory responses.

Similarly, the active monomer combination (10 μM ellagic acid + 1 μM maslinic acid) exhibited superior efficacy in cytokine regulation (Figure 3E–H). This combination reduced TNF-α, IL-1β, and IL-6 levels by 81.72 pg/mL, 31.16 pg/mL, and 574.88 pg/mL, respectively, while elevating IL-10 production by 88.16 pg/mL. Notably, the monomer combination achieved more pronounced cytokine modulation than individual compounds, confirming the synergistic anti-inflammatory effects predicted through combination index (CI) analysis.

These findings confirm that both the crude extract and active monomer combinations target NF-κB-mediated cytokine networks, with synergistic effects arising from the complementary modulation of pro- and anti-inflammatory pathways. The multi-cytokine regulatory capacity aligns with the network pharmacology predictions of multi-target therapeutic mechanisms, further supporting the therapeutic potential of pomegranate peel–hawthorn formulations in UC management.

### 3.4. Effects of Different Drugs or Combinations on DSS-Induced UC Mice

**Figure 4:**
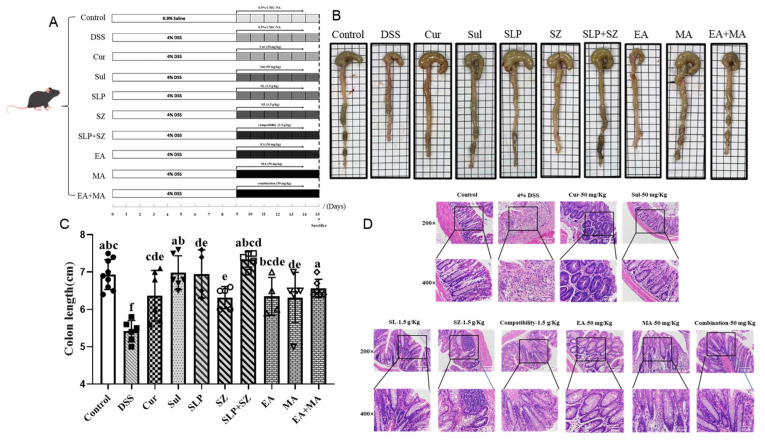
Animal protocols and pathological changes in the colon. Animal protocols (**A**), pathological changes (**B**), colon length (**C**), and HE staining of colon (**D**). Cur, curcumin; SLP, pomegranate peel extract; SZ, hawthorn extract; EA, ellagic acid; MA, maslinic acid. Different letters indicate *p* < 0.05.

A post-experimental analysis of colonic morphology revealed significant pathological changes in DSS-induced UC mice. As shown in Figure 4B,C, UC mice exhibited marked colonic shortening (5.42 cm vs. normal group: 6.93 cm), with atrophic changes, mucosal erosions, and ulcerative lesions, accompanied by loose, unformed stools. All treatment groups showed varying degrees of improvement, with the combination groups—pomegranate peel–hawthorn extract (SLP+SZ) and ellagic acid–maslinic acid (EA+MA)—demonstrating superior efficacy in restoring colonic integrity, resolving erosions/ulcers, and normalizing stool consistency.

A histopathological evaluation of distal colon sections (1–2 cm from the anus) via H&E staining (Figure 4D) confirmed these observations. Normal mice displayed intact epithelial barriers, a well-preserved crypt architecture, and no inflammatory infiltration. By contrast, DSS-induced UC mice exhibited distorted and atrophic crypts, epithelial barrier disruption, goblet cell depletion, and dense plasma cell infiltration between crypt bases and the muscularis mucosa. All treatment groups mitigated these pathological features, with the SLP+SZ and EA+MA groups showing near-complete restoration of epithelial integrity. These combination therapies significantly reduced inflammatory cell infiltration, regenerated goblet cells, and restored U-shaped crypt morphology (Figure 4D), indicating robust protection against UC-associated tissue damage.

These findings corroborate the therapeutic superiority of combinatorial formulations in preserving colonic structural integrity and resolving UC-associated histopathological alterations, consistent with their observed anti-inflammatory efficacy in cellular assays.

### 3.5. Comprehensive Therapeutic Effects on Systemic Inflammation, Oxidative Stress, and Organ Function in DSS-Induced UC Mice

**Figure 5:**
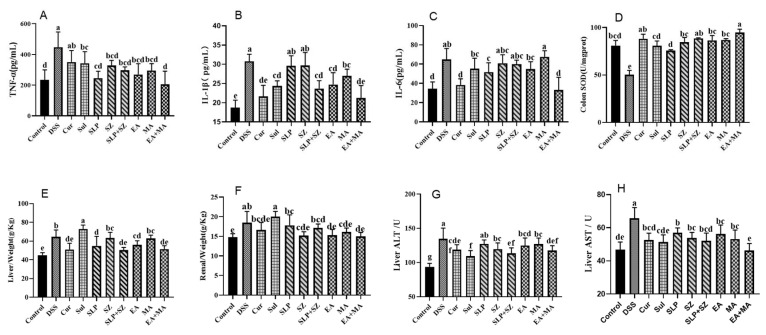
Changes in physiological and biochemical indicators in a DSS-induced UC mice model. TNF-α (**A**), IL-1β (**B**), and IL-6 (**C**) contents of different drugs or combinations; SOD (**D**), ALT (**G**), and AST (**H**) activities of different drugs or combinations; liver (**E**) and renal (**F**) indices of different drugs or combinations. Different letters indicate *p* < 0.05.

Therapeutic interventions were evaluated for their systemic anti-inflammatory, antioxidant, and organ-protective effects in DSS-induced UC mice. Serum cytokine analysis (Figure 5A–C) demonstrated that DSS challenge significantly elevated pro-inflammatory mediators (TNF-α (446.52 vs. 234.20 pg/mL), IL-1β (30.78 vs. 18.70 pg/mL), and IL-6 (64.86 vs. 34.55 pg/mL)) compared with healthy controls. The ellagic acid–maslinic acid (EA+MA) combination showed superior anti-inflammatory efficacy, normalizing cytokine levels (TNF-α: 204.78; IL-1β: 21.23; IL-6: 33.19 pg/mL) and outperforming individual treatments and crude extract combinations (SLP+SZ).

Oxidative stress assessment revealed DSS-induced colonic SOD depletion (50.10 vs. 80.94 U/mg·prot in controls), indicating severe oxidative damage (Figure 5D). EA+MA restored SOD activity most effectively, correlating with reduced histopathological injury, confirming dual anti-inflammatory and antioxidant mechanisms.

Systemic physiological evaluations identified UC-associated hepatic edema (increased liver weight-to-body ratio, *p* < 0.01) without renal involvement (Figure 5E,F). The positive control (Sul) exacerbated hepatic metabolic burden, while both SLP+SZ and EA+MA combinations attenuated liver injury markers (ALT: 98.74/102.35 vs. 134.65 U/L; AST: 51.62/54.13 vs. 65.78 U/L) (Figure 5G,H) without worsening hepatic edema.

These findings highlight the multi-dimensional therapeutic benefits of combinatorial formulations, effectively suppressing systemic inflammation, restoring redox balance, and protecting against UC-associated hepatotoxicity, aligning with their predicted multi-target mechanisms.

### 3.6. Mechanisms of Different Drugs or Combination Therapies in the Treatment of UC

**Figure 6:**
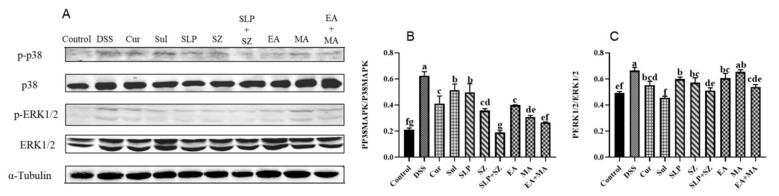
Expression of MAPK pathway-related proteins of different drugs or combinations. Western blot (**A**), p-P38/P38 ratio (**B**), and p-ERK_1/2_/ERK_1/2_ ratio (**C**). Different letters indicate *p* < 0.05.

The MAPK pathway acts as an upstream pathway of the NF-κB pathway but is also a key signaling pathway in promoting inflammatory responses. As previously demonstrated, the combined treatment could more effectively inhibit the activation of NF-κB, thereby reducing the transcription of various pro-inflammatory mediators and cytokines. Compared with the blank group mice, in the DSS group mice, the ratios of p-p38 to p38 and p-ERK1/2 to ERK1/2 in the colonic tissues significantly increased due to continuous ulcerative colitis, indicating the activation of the inflammatory pathway. After treatment with the combination of pomegranate peel and hawthorn, as well as ellagic acid combined with hawthorn acid, the ratios of p-p38 to p38 and p-ERK1/2 to ERK1/2 were significantly reduced. The inhibitory effect was comparable to that of the positive drug group and tended to return to a normal state (Figure 6A–C). These experimental results suggest that the combined treatment can intervene in treating ulcerative colitis through multiple pathways and factors, thus protecting the colon from further damage.

### 3.7. Effects of Different Drugs or Combinations on the Gut Microbiota of DSS-Induced UC Mice

**Figure 7:**
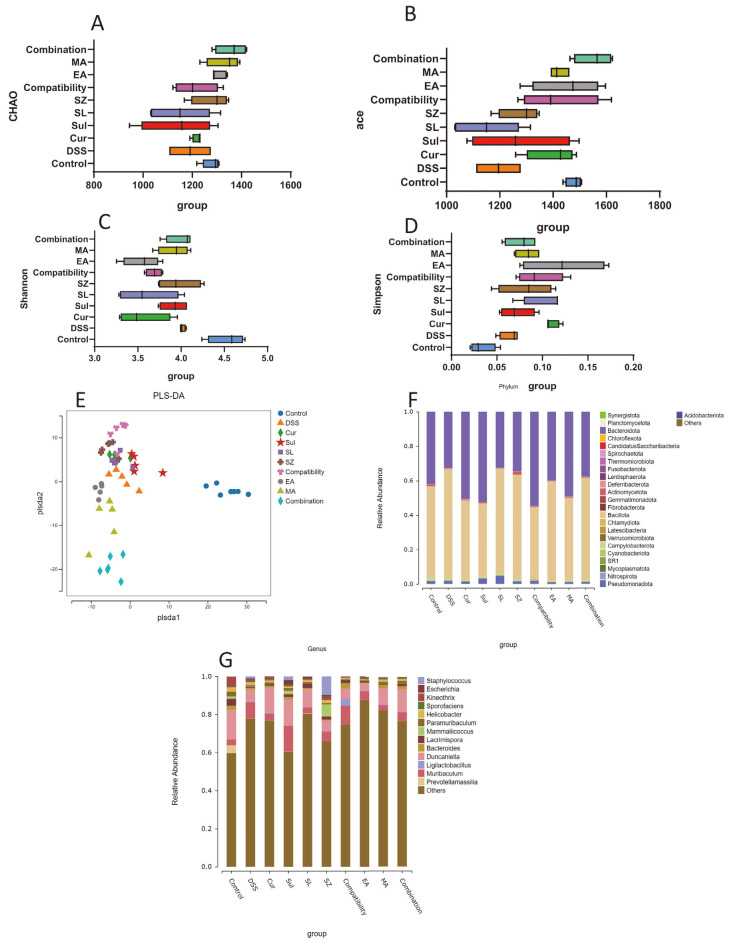
Effects of combination drugs on the gut microbiota of DSS-induced UC mice. Alpha diversity analysis of Chao1 (**A**), ACE (**B**), Shannon (**C**), and Simpson (**D**) indices. Effect of combination of drugs on the beta diversity of intestinal flora in mice with colitis (**E**). Stacked diagram of the distribution of phylum-level flora (**F**). Stacked map of genus-level colony distributions (**G**).

A gut microbiota analysis (Figure 7A–D) revealed significantly reduced alpha diversity in DSS-induced colitis mice compared with healthy controls. The Chao1 and ACE indices indicated diminished microbial richness, while decreased Shannon and Simpson indices reflected impaired diversity and uneven species distribution. Combinatorial treatments (SLP+SZ and EA+MA) effectively restored microbial homeostasis, with the Chao1 and ACE indices recovering to levels comparable to the controls (*p* > 0.05) and the Shannon/Simpson indices approaching normal values, suggesting rebalanced diversity and evenness.

PLS-DA (Figure 7E) highlighted distinct clustering between the DSS and control groups, confirming compositional shifts in fecal microbiota. The treated groups exhibited scattered distribution near DSS clusters, indicating partial restoration of the microbial profiles.

At the phylum level (Figure 7F), DSS mice showed decreased *Bacteroidota* and increased *Bacillota* abundance compared with the controls. Combinatorial therapies normalized these phyla closer to healthy levels, outperforming monotherapies. At the genus level (Figure 7G), the DSS mice displayed elevated *Muribaculum* and reduced *Duncaniella*, *Lacrimispora*, and *Kineothrix* abundances. Drug treatments partially reversed these dysbiotic trends. Collectively, combinatorial interventions mitigated colitis-associated microbial imbalances, correlating with their anti-inflammatory and tissue-protective efficacy.

LEfSe analysis revealed significant microbiota alterations between the DSS-induced UC mice and the treatment groups (Figures S7 and S8). The combinatorial herbal formulation (compatibility group) markedly increased the abundance of *Lactobacillaceae*—a probiotic family critical for gut health and immune modulation—with a linear discriminant analysis (LDA) score > 4.0 (Figure S7). This enrichment correlated with improved intestinal barrier parameters observed in histopathological assessments. Concurrently, the active monomer combination (EA+MA) significantly elevated *Terrisporobacter* levels (LDA score = 3.8), a genus renowned for producing short-chain fatty acids (SCFAs), including acetate, propionate, and butyrate (Figure S8). These metabolites are essential for maintaining colonic pH homeostasis, enhancing epithelial integrity, and exerting anti-inflammatory effects. The DSS-induced UC mice exhibited pathogenic microbial shifts characterized by an overgrowth of *Rikenella* (LDA > 3.2) and *Gehongia* (LDA > 3.2), genera associated with mucosal degradation and dysbiosis. Therapeutic interventions reversed these trends, suppressing pathobionts while restoring SCFA-producing commensals. These findings demonstrate that combinatorial therapies reshape gut microbial ecology by enriching beneficial taxa (*Lactobacillaceae*; *Terrisporobacter*) and suppressing inflammation-associated genera (*Rikenella*; *Gehongia*), providing a microbial basis for their observed therapeutic efficacy in UC management.

## 4. Conclusions

This integrative investigation demonstrated that pomegranate peel–hawthorn combinations and their active constituents exert synergistic therapeutic effects in UC through multi-modal mechanisms, encompassing NF–κB/MAPK pathway inhibition, oxidative stress mitigation, and gut microbiota restoration. Bridging traditional herbal medicine with modern pharmacology approaches, this study provides a paradigm for developing plant-derived combinatorial therapies against complex inflammatory disorders.

## Data Availability

The data are available from the authors upon request.

## Supplementary Materials

The following supporting information can be downloaded at https://www.mdpi.com/article/10.3390/cimb47040243/s1.
