# Supplementary material for: Synergistic Anti-Inflammatory Effects of Pomegranate Peel–Hawthorn Combinations in Ulcerative Colitis: Network Pharmacology Prediction and Experimental Validation
Source: Curr Issues Mol Biol. 2025 Apr 1;47(4):243. doi: 10.3390/cimb47040243 (PMC12025673; doi:10.3390/cimb47040243)
Supplement: Supplementary file 1 [file cimb-47-00243-s001.zip › cimb-3551360-supplementary.docx]

**Synergistic Anti-Inflammatory Effects of Pomegranate Peel-Hawthorn Combinations in Ulcerative Colitis: Network Pharmacology Prediction and Experimental Validation**

Shouqing Zhang a, Quanyuan Qiu a，Mengzhen Yuan a, Jiajia Yu a, Weiwei Gao a, Xi Wang a, Zhen Liu a,Peng Yu a and Cen Xiang a,*,Yuou Teng a,*

^1^ China International Science and Technology Cooperation Base of Food Nutrition/Safety and Medicinal Chemistry, State Key Laboratory of Food Nutrition and Safety, Tianjin University of Science and Technology, Tianjin, 300457, China.

**CONTENT**

[Table S1 Pomegranate peel active ingredient search 1](#_Toc14112)

[Fig. S1 Pomegranate peel-Active Ingredient-Target-Ulcerative Colitis Network Diagram Analysis 2](#_Toc1388)

[Fig. S2 Hawthorn-Active Ingredient-Target-Ulcerative Colitis Network Diagram Analysis 3](#_Toc19873)

[Fig. S3 Protein interaction network diagram of Pomegranate peel 4](#_Toc18660)

[Fig. S4 Protein interaction network diagram of Hawthorn 5](#_Toc21474)

[Fig. S5 Gene Ontology Analysis of Pomegranate peel 6](#_Toc4935)

[Fig. S6 Gene Ontology Analysis of Hawthorn 7](#_Toc28653)

[Fig. S7 Lefse Analysis of Hawthorn（SZ）, Pomegranate peel (SLP) and Compatibility 8](#_Toc5548)

[Fig. S8 Lefse Analysis of Ellagic acid, Maslinic acid and Compatibility 9](#_Toc3278)

#

# Table S1 Pomegranate peel active ingredient search

| TCM | MOL-ID | Activity molecular |
| --- | --- | --- |
| pomegranate peel | MOL009274 | Fritillaziebinol |
|  | MOL001002 | ellagic acid |
|  | MOL000492 | (+)-catechin |
|  | MOL000422 | kaempferol |
|  | MOL000358 | beta-sitosterol |
|  | MOL000006 | luteolin |
| hawthorn | MOL000422 | kaempferol |
|  | MOL000359 | sitosterol |
|  | MOL000449 | Stigmasterol |
|  | MOL000449 | Maslinic acid |


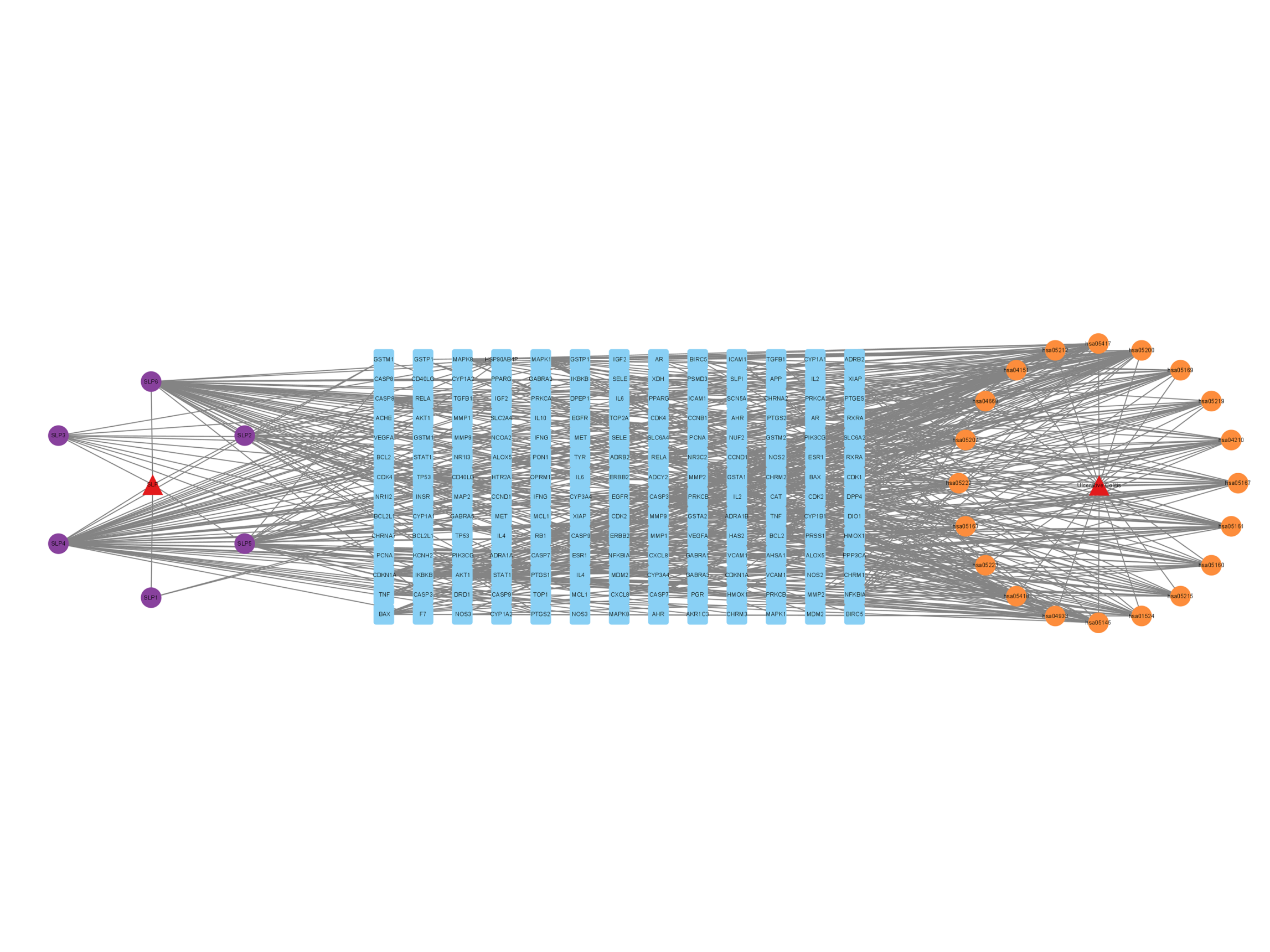


# Fig. S1 Pomegranate peel-Active Ingredient-Target-Ulcerative Colitis Network Diagram Analysis


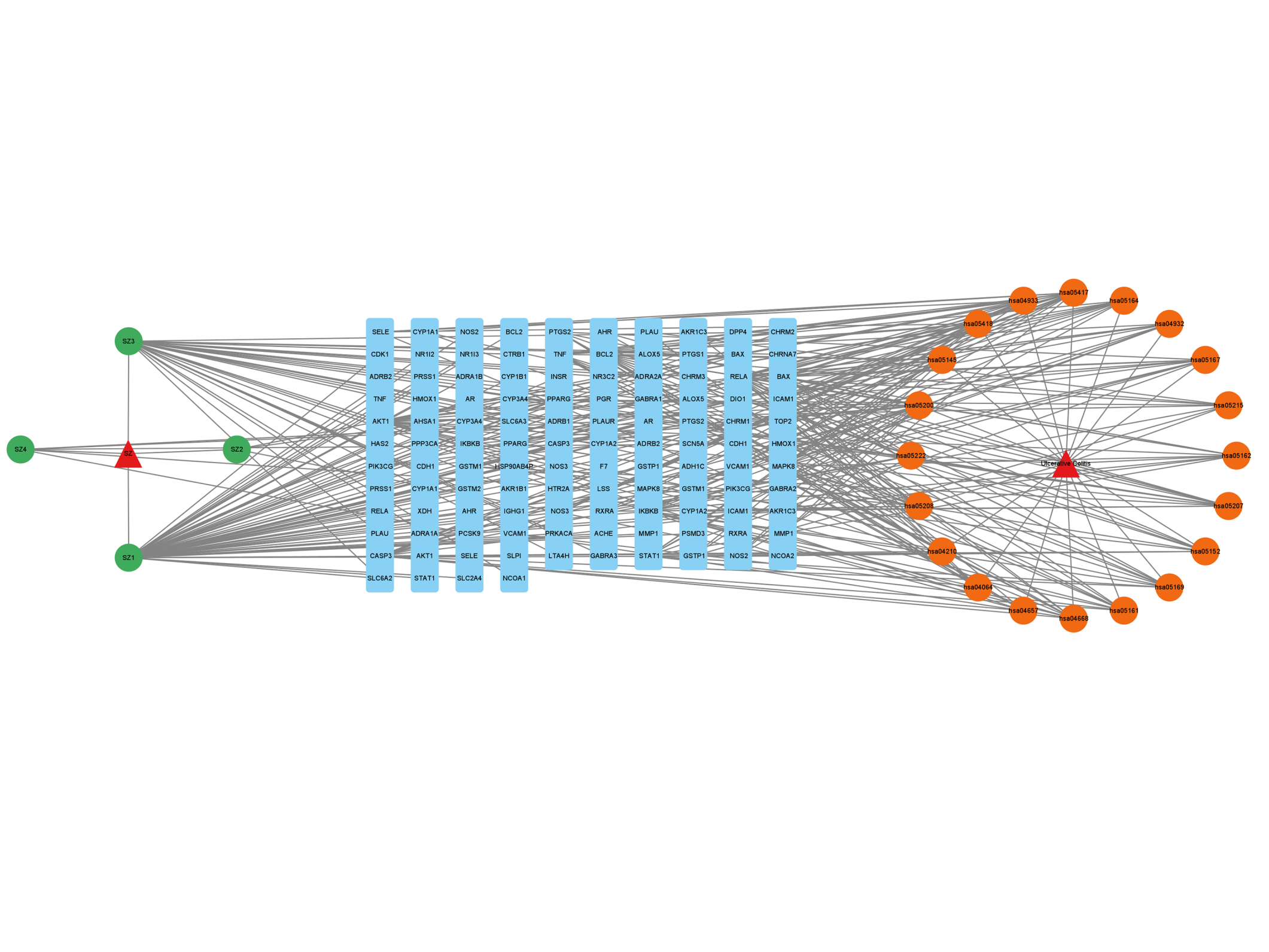


# Fig. S2 Hawthorn-Active Ingredient-Target-Ulcerative Colitis Network Diagram Analysis


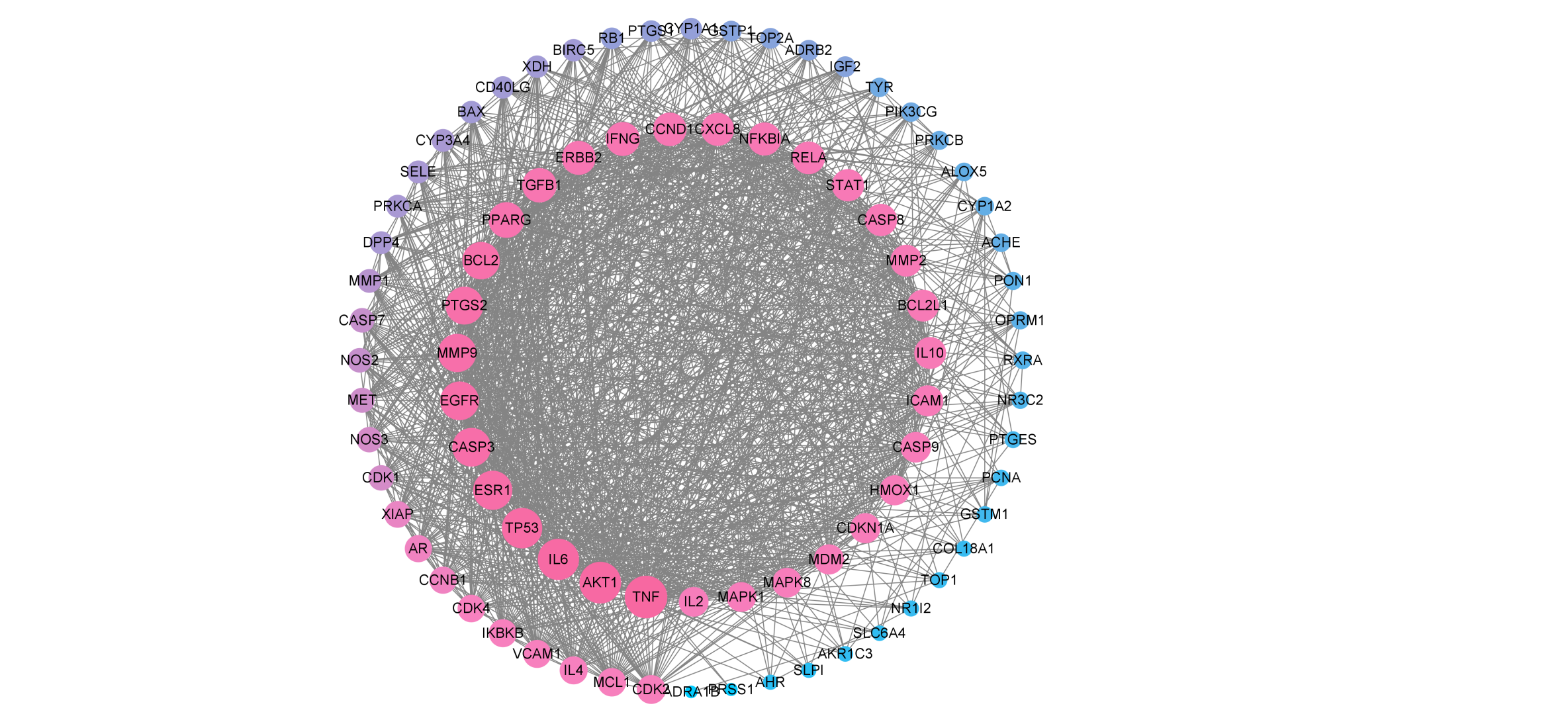


# Fig. S3 Protein interaction network diagram of Pomegranate peel


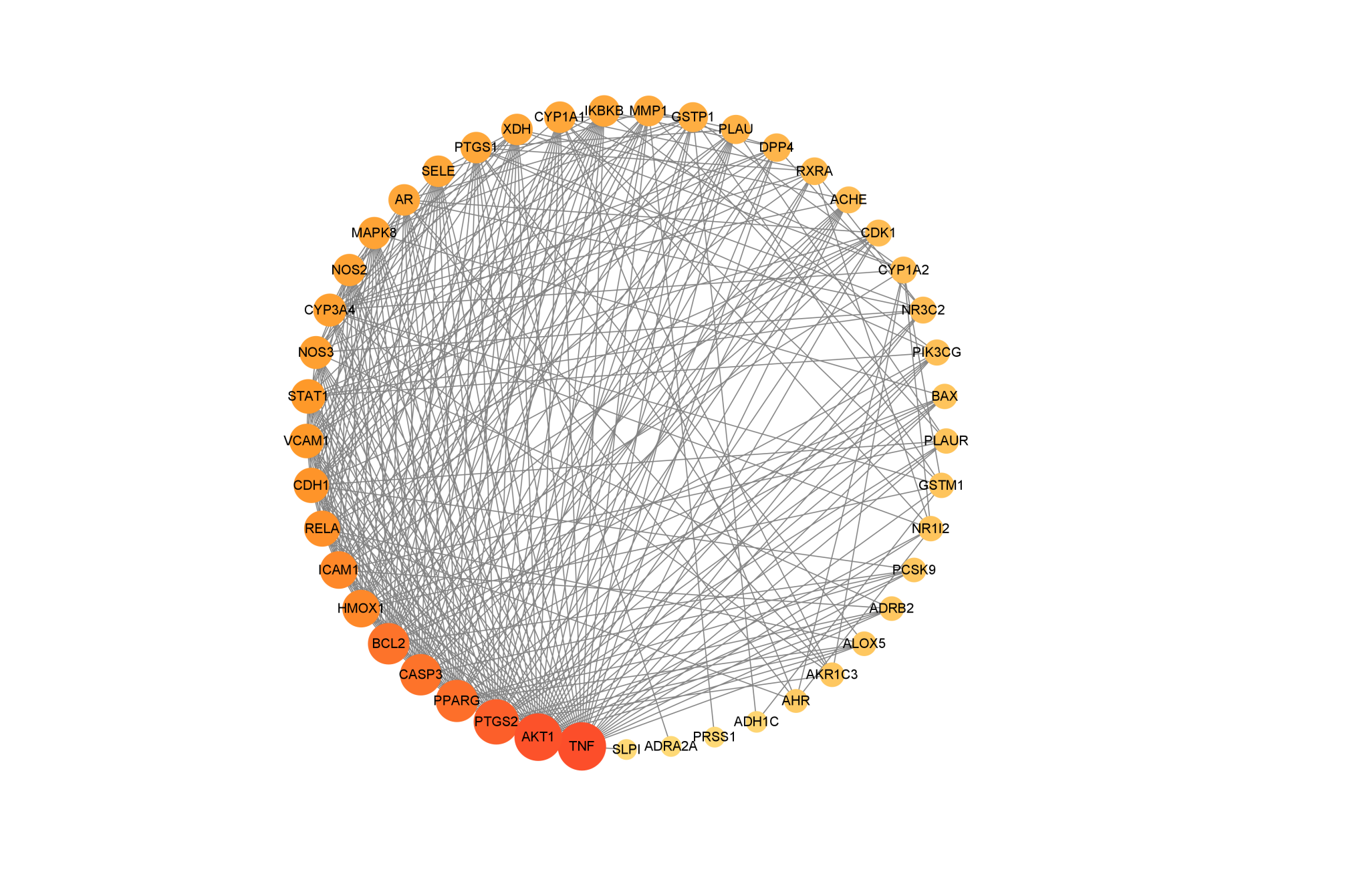


# Fig. S4 Protein interaction network diagram of Hawthorn


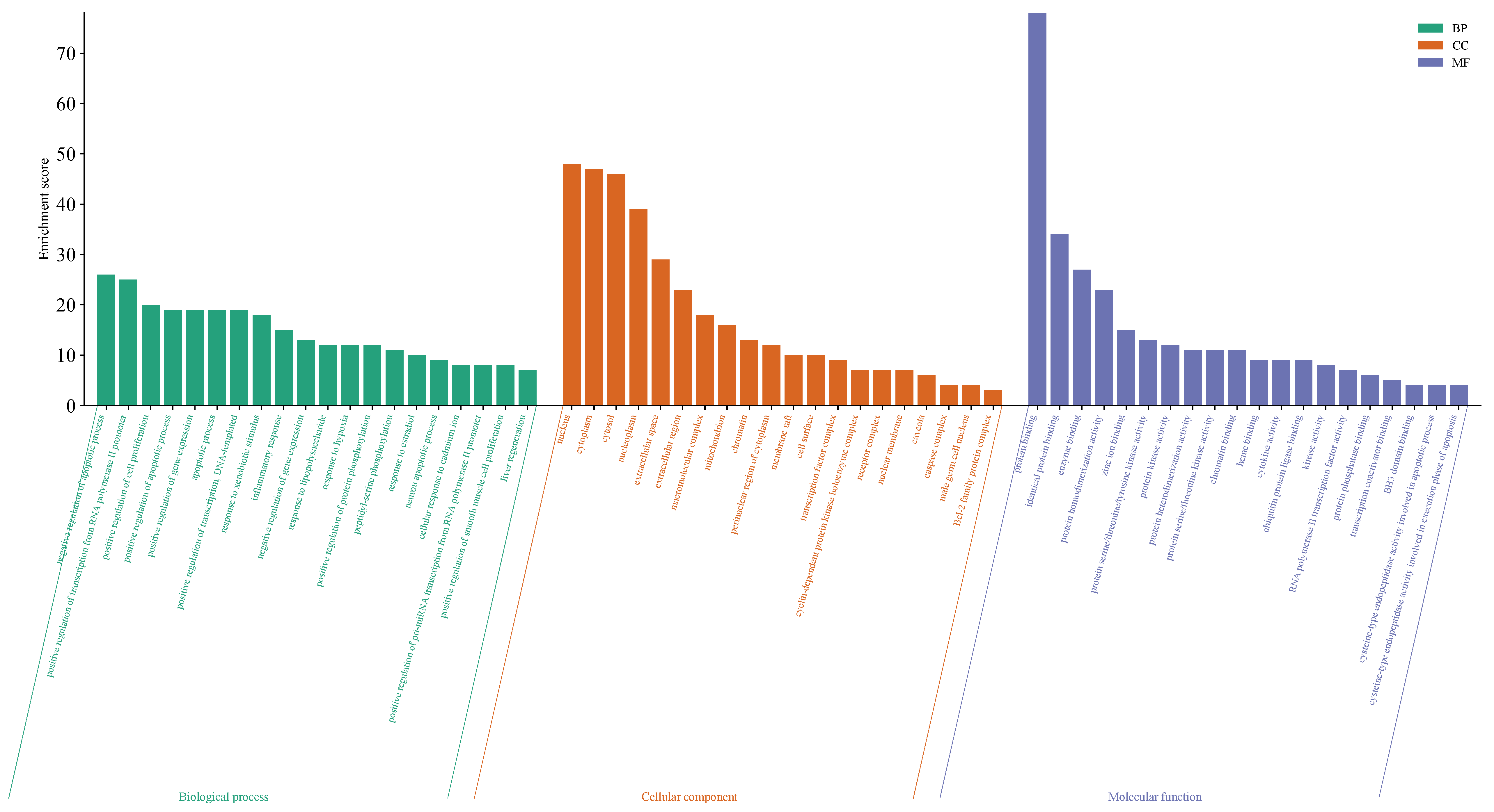


# Fig. S5 Gene Ontology Analysis of Pomegranate peel


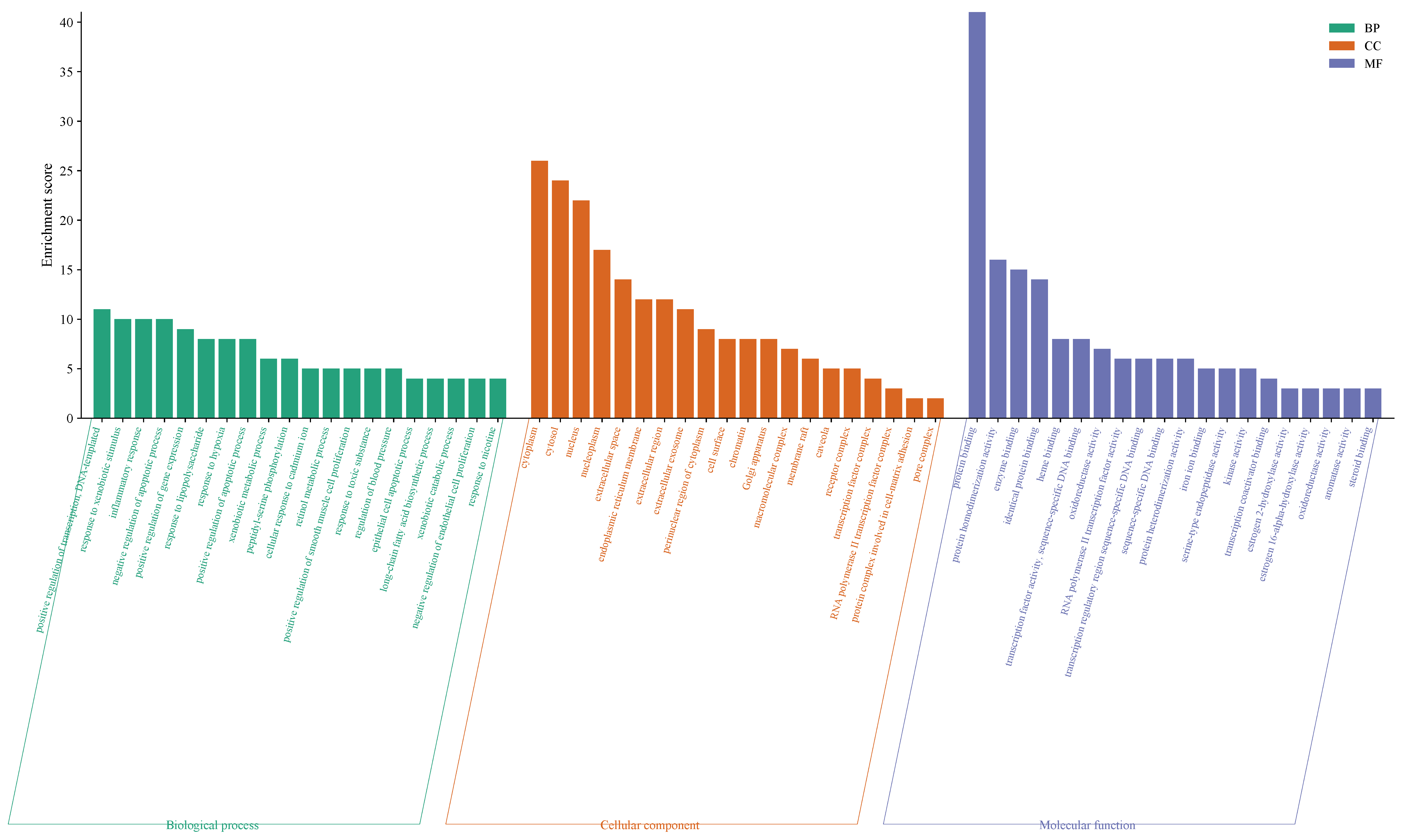


# Fig. S6 Gene Ontology Analysis of Hawthorn


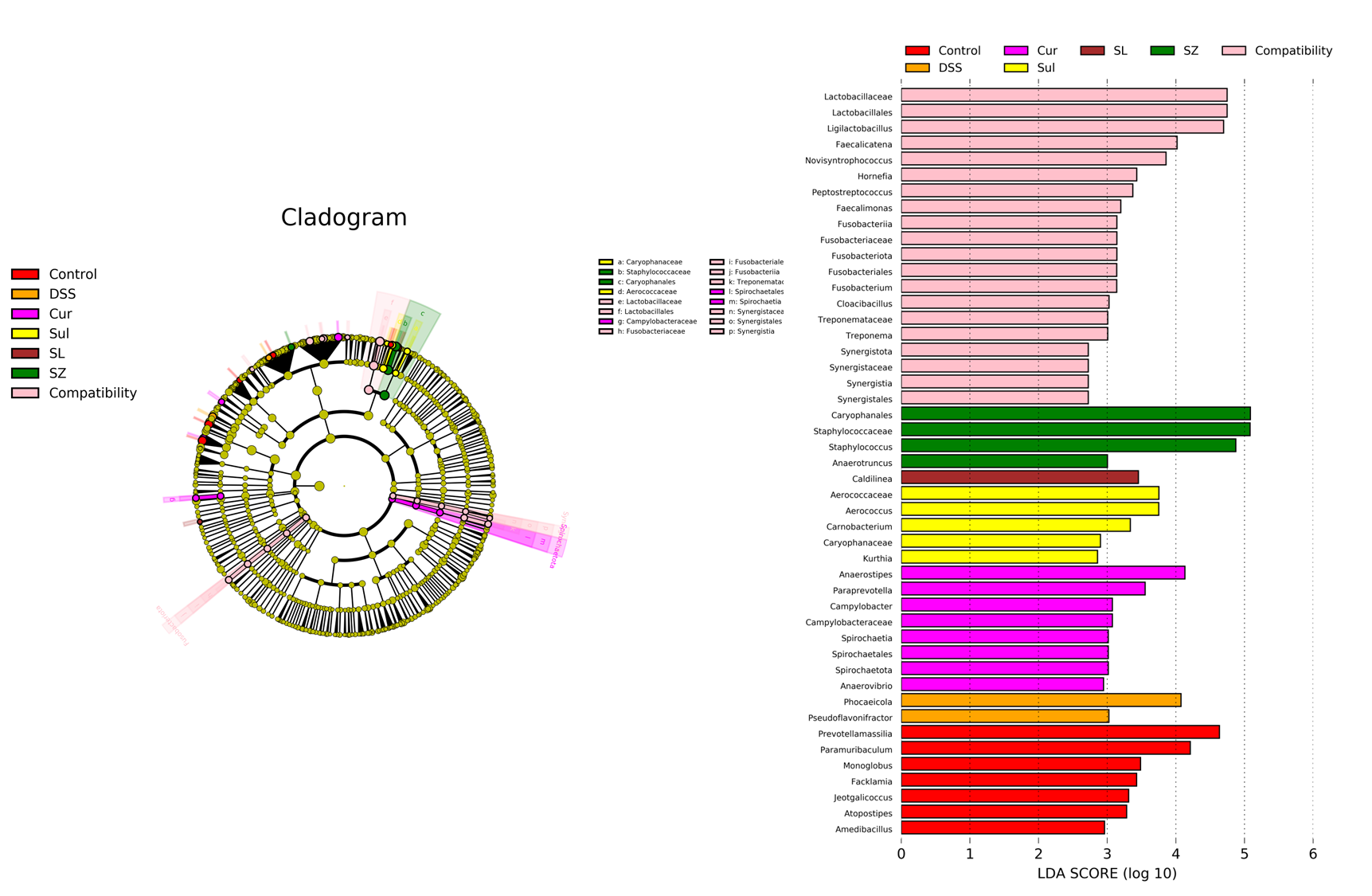


# Fig. S7 Lefse Analysis of Hawthorn（SZ）, Pomegranate peel (SLP) and Compatibility


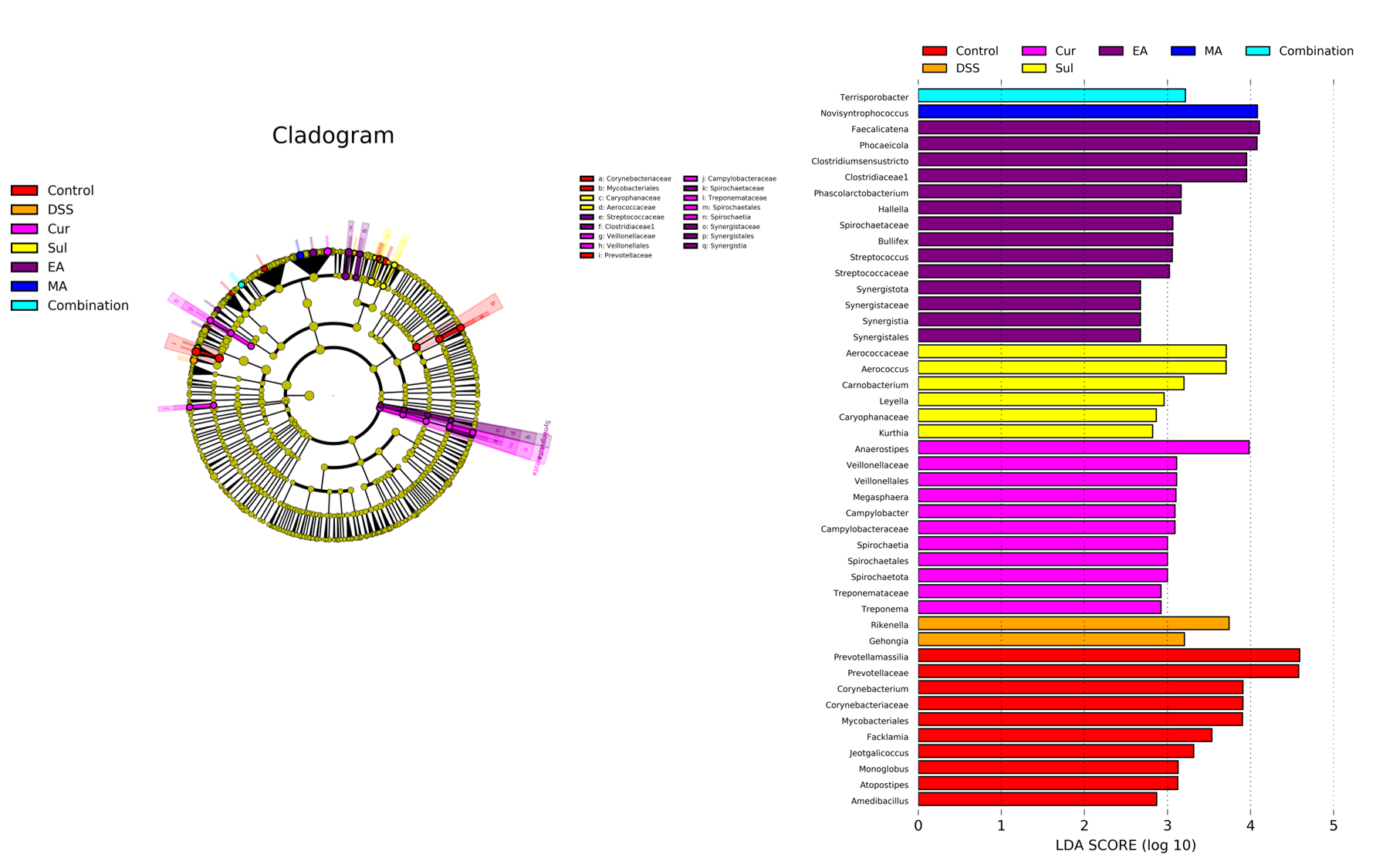


# Fig. S8 Lefse Analysis of Ellagic acid, Maslinic acid and Compatibility
